# Clinical evaluation of traditional Chinese medicine on mild active ulcerative colitis: A multi-center, randomized, double-blind, controlled trial

**DOI:** 10.1097/MD.0000000000021903

**Published:** 2020-08-28

**Authors:** Fu-Shun Kou, Lei Shi, Jun-Xiang Li, Zhi-Bin Wang, Rui Shi, Tang-You Mao, Xiao Ke, Bei-Ping Zhang, Xiao-Jun Yang, Xin-Li Wen, Wei-Yang Zheng, Xiao Han, Pang-Hua Ding, Jun Dong

**Affiliations:** a Graduate School, Beijing University of Chinese Medicine, No. 11, North Third Ring East Road, Chaoyang District; b Gastroenterology Department, Dongfang Hospital, Beijing University of Chinese Medicine, No. 6, 1st Section, Fangxingyuan, Fangzhuang, Fengtai District; c School of Life Sciences, Beijing University of Chinese Medicine, No. 11, North Third Ring East Road, Chaoyang District, Beijing; d Department of Spleen and Stomach Diseases, The Second People's Hospital affiliated to Fujian University of Traditional Chinese Medicine, 282 Wusi Lu, Fuzhou City, Fujian Province; e Gastroenterology Department, The Second Affiliated Hospital of Guangzhou University of Traditional Chinese Medicine, No. 111 Dade Road, Yuexiu District, Guangzhou; f Department of Gastroenterology, Chongqing Hospital of Traditional Chinese Medicine, No. 6, the 7th branch of Panxi road, Jiangbei District, Chongqing; g Department of Gastroenterology, Shanxi Hospital of Traditional Chinese Medicine, No. 4, Xihuamen, Lianhu District, Xi’an Shanxi Province; h Department of Gastroenterology, Peking Union Medical College Hospital, No. 1 shuaifuyuan, Dongdan, Dongcheng District, Beijing; i Central laboratory Department, Jiangsu Province Hospital of Chinese Medicine, Affiliated Hospital of Nanjing University of Chinese Medicine, No. 155, Hanzhong Road, Gulou District, Nanjing City, Jiangsu Province, PR China.

**Keywords:** traditional Chinese medicine, Hudi enteric-coated capsules, mild active ulcerative colitis, double-blind, double-dummy, randomized controlled trial

## Abstract

**Introduction:**

Ulcerative colitis (UC) is a chronic inflammatory bowel disease (IBD) characterized by a relapsing-remitting course owing to recurrent intestinal inflammation. UC often has symptoms such as intermittent rectal bleeding, diarrhea, and abdominal pain. As the precise etiology of UC has not completely clarified, UC has become a public health challenge worldwide. According to an epidemiological survey, there were about 350,000 new cases of IBD in China from 2005 to 2014. By 2025, the number of IBD patients in China will reach 1.5 million. Traditional Chinese medicine (TCM) has been widely used to treat UC in China, however, it is still challenging to systematically determine the efficacy of in UC. Therefore, this trial aims to evaluate the clinical efficacy and safety of CHM in the treatment of mild active UC patients.

**Methods:**

A multi-center, double-blinding, double-dummy, active-controlled, randomized trial will be established. A total of 240 patients in 6 centers with mild active UC (Mayo score is 3–5 points) and TCM syndrome of damp-heat stasis blocking and spleen-qi deficiency will be randomly allocated in the ratio of 1:1 to 2 groups: the experimental group and the control group. The experimental group will receive Hudi enteric-coated capsules (HEC) and enteric-coated mesalazine tablets placebo; the control group will receive enteric-coated mesalazine tablets and HEC placebo. Each group will be treated for 8 weeks. The primary therapeutic outcome: the rate of clinical efficacy and clinical remission at 8 weeks of treatment (last survey point) according to the modified Mayo score. The secondary outcomes: individual symptom score, TCM syndrome score, endoscopic response rate, mucosal healing rate, and quality of life scale score. Outcomes will be assessed at baseline and the end of the trial. Besides, intestinal mucosa, stools and blood biopsies from the mild active UC patients before and after treatment will be collected to reveal the underlying mechanisms.

**Discussion:**

The results of this trial will provide compelling evidence of the efficacy and safety of HEC for treatment of mild active UC and preliminarily show the potential mechanism of how HEC acts. Finally, it will widen treatment options for patients with mild active UC.

## 1 Introduction

Ulcerative colitis (UC) is a kind of inflammatory bowel diseases (IBD) characterized by chronic nonspecific inflammatory colorectal disease with a yet-uncharacterized etiology. UC often has symptoms such as intermittent rectal bleeding, diarrhea, and abdominal pain. It mainly affects young and middle-aged people between 18 and 50 years old.^[1]^ UC-related colorectal cancer accounts for 9% to 11% of the death causes of UC patients.^[2]^ UC was mainly prevalent in developed countries of Europe and North America. Studies have reported that the annual incidence of UC varies from 8.8 to 23.14 per 100,000 person-years in North America, 1.7 to 57.9 per 100,000 person-years in Europe, and prevalence was 139.8 to 286.3 per 100,000, and 2.42 to 412.0 per 100,000, respectively.^[3]^ According to an epidemiological survey, there were about 350,000 new cases of IBD in China from 2005 to 2014, and by 2025, the amount of IBD patients in China will reach 1.5 million.^[4]^ As the precise etiology of UC has not completely clarified, UC has become a public health challenge worldwide,^[5]^ and because of the widespread of the mild active period, our trail will focus on this specific type.

UC is treated with 5-aminosalycilate acid (5-ASA), corticosteroids, immune suppressants, and biological agents currently, especially, 5-ASA is recommended as a first-line therapy for mild-to-moderate UC patients.^[6]^ But those medicine often result in different adverse effects, a low response rate and a high risk of infection,^[5]^ however, complementary and alternative medicine (CAM), like traditional Chinese medicine (TCM), with lower adverse effects were used seldom in UC. TCM is one of the most commonly used modality for UC patients, accounting for 19% to 54% of CAM users,^[7]^ that is been known as useful in improving symptoms, reducing medical expenses and clinical evaluation for UC patients, but it is still challenging to systematically determine the efficacy of TCM in treatment.^[8]^

Numerous studies based on UC mouse models and clinical trials, especially randomized double-blinding clinical trials, remains the gold standard for evaluating the efficacy of TCM.^[9]^ To obtain more conclusive results on the risk-benefit ratio of TCM for UC, it is necessary to design and conduct a high-quality clinical trial with appropriate blinding method and a large number of patients. Hudi enteric-coated capsules (HEC) has finished a multi-center clinic randomized controlled trial in China,^[10]^ it confirmed that HEC played a pivotal role in treating mild to moderate UC by relieving symptoms effectively, but as the shortage of colonoscopy examination and treatment time, this trial was not rigorous enough. Besides, the mechanism of HEC in treating UC was not clear.

Therefore, the primary aim of this multi-center, double-blinding, double-dummy, randomized controlled trial is to evaluate the clinical efficacy and safety of HEC in the treatment of mild active UC patients. Meanwhile, we want to reveal the underlying mechanism of HEC how to treat mild active UC by molecular biology technology from the clinic specimens.

## 2 2.Materials and methods

### 2.1 Ethical approval and consent to participate

The study is conducted in accordance with the Declaration of Helsinki (Edinburgh 2000 version). The final protocol (Date: April 15, 2019) of this trial has been approved by the team leader unit, Research Ethical Committee of Jiangsu Province Hospital of Chinese Medicine (Version number: 2019NL-039-02). In addition, this trial has been registered in the Chinese Clinical Trial Registry (No. ChiCTR1900023158, registered May 14, 2019). Before the enrollment, all participants would sign written informed consents. If there will be any significant modification of the protocol, it should be reviewed by the research ethical committee and updated on the registry web (http://www.chictr.org.cn) in time. Each participating center has conducted ethical filing and has the ethical approval of the leader unit. All volunteers signed the informed consent form before the trial and details could be inquired in the registry web or the corresponding author.

### 2.2 Trial design

**Figure 1:**
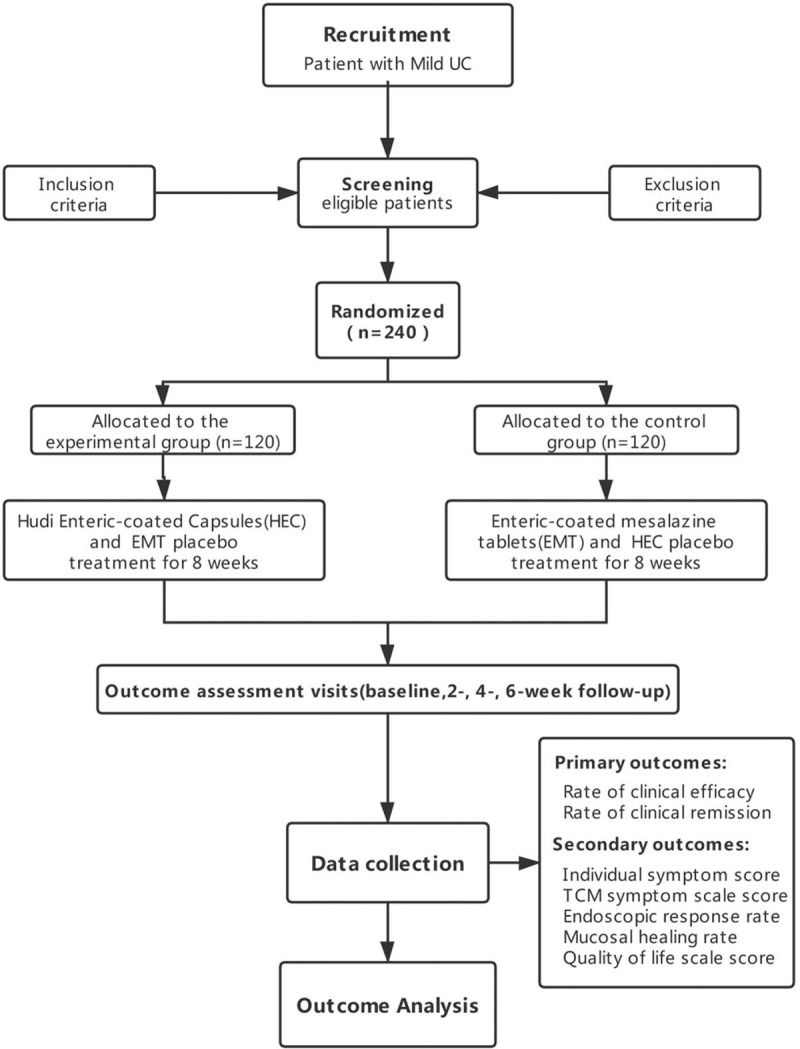
Flowchart of the trial design. TCM = traditional Chinese medicine, ALT = alanine aminotransferase, AST = aspartate aminotransferase, TBIL = total bilirubin, DBIL = direct bilirubin, γ-GT = γ-glutamyl transpeptidase, ALP = alkaline phosphatase, Scr = serum creatinine, BUN = blood urea nitrogen, HEC = Hudi enteric-coated capsules, EMT = enteric-coated mesalazine tablets.

This study is a multi-center, double-blind, double-dummy, randomized, parallel positive controlled trial. We adopted a noninferiority trial design and followed the Standardized Protocol Interventions: Recommendations for Interventional Trials (SPIRIT) 2013 Statement.^[11]^ The experimental group was treated with HEC and enteric-coated mesalazine tablets (EMT) placebo, while HEC placebo and EMT were used in the control group. A total of 240 patients diagnosed with mild active ulcerative colitis (MAUC) and TCM syndrome of damp-heat stasis blocking and spleen-qi deficiency in 6 clinical trial centers are eligible for enrolment. We will implement randomization with a 1:1 allocation ratio in the experimental group (n = 120) or the control group (n = 120), and each patient will undergo an 8-week treatment. The study flowchart is showed in Figure 1.

### 2.3 Number of Samples

Number of Samples was determined based on the calculation of noninferiority trial design. The efficacy rates of EMT ranges from 65% to 71%,^[12,13]^ and the average rate is about 68%. Our previous study found that the efficacy rates of HEC is 83.11% in treating MAUC. The sample size was calculated by the following formula: 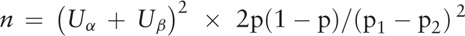


*U*α, *U*β is the corresponding *U* value of α, β. When α equaling to 0.05, β equaling to 0.1, *U*α (0.05) is 1.645, *U*β (0.2) is 0.842. *P*_1_ and *P*_2_ represent the efficacy rates of HEC and EMT. *P* equals to (*P*_1_ + *P*_2_) /2 × 100%. The certainty was set at 80%, considering 20% of sample lose. The sample number was set to 120 for each group, 240 in total, and 40 samples in each center.

### 2.4 Study setting

A total of 240 MAUC patients were recruited by 6 clinical trial centers in China from June 2019 to December 2021. Six clinical trial centers were listed as follows: Jiangsu Provincial Hospital of Chinese Medicine, Peking Union Medical College Hospital of Chinese Academy of Medical Sciences, Guangdong Provincial Hospital of Chinese Medicine, Chongqing Traditional Chinese Medicine Hospital, The Second Affiliated Hospital of Fujian Traditional Chinese Medicine University, and Shaanxi Traditional Chinese Medicine Hospital. The inclusion of the case complies with the principle of geographical balance nationwide. Internal physicians will screen eligible patients from outpatient clinics. Before the enrollment, all patients will receive a comprehensive view of informed consent, including objective, interventions, scheduling, the benefits and risks of this trial. An informed consent form is mandatory for enrollment.

### 2.5 Eligibility criteria

Diagnostic criteria:

1. The western medicine diagnostic criteria of UC will refer to Chinese consensus on diagnosis and treatment of IBD (Beijing, 2018).^[14]^
2. The TCM diagnostic criteria of the damp-heat stasis blocking and spleen-qi deficiency syndrome will refer to the Clinic terminology of traditional Chinese medical diagnosis and Consensus of traditional Chinese medical in diagnosis and treatment of UC.^[15,16]^ Diagnosis of this syndrome including the main and minor symptoms as following:

Main symptoms:

1. Diarrhea, mucus and bloody stools;
2. Abdominal pain;
3. Tesmus;
4. Tired and lazy;

Minor symptoms:

1. burning anus;
2. short and dark-color urine;
3. dry and bitter in the mouth;
4. drowsiness of limbs;
5. poor appetite.

Tongue and pulse: red tongue, thick yellow fur with tooth marks on the sides, and smooth pulse.

Confirmation of the syndrome type: 2 main symptoms (required for the first item) plus 2 minor symptoms, which can be diagnosed by referring to the tongue and pulse.

Inclusion criteria:

1. Diagnosed with active UC.
2. Defined by modified Mayo score of 3 to 5 points (mild period).^[14]^
3. Differentiation of TCM syndrome is damp-heat stasis blocking accompanied with spleen-qi deficiency.
4. Age >18 and <65 years.
5. Voluntary to participate and providing written informed consents, as required by Good Clinical Practice.

Exclusion criteria:

1. Patients during remission period, moderate or severe period.
2. Pregnancy, lactation, or have desire for pregnancy.
3. Severe allergy or allergic to mesalazine or any of the ingredients in HEC.
4. Have cardiovascular, pulmonary, liver, renal, endocrine, nerve, and hematology diseases. Blood creatinine level, blood alanine transaminase or aspartate aminotransferase level above the upper limit of normal.
5. Have serious complications, such as intestinal stenosis, intestinal obstruction, intestinal perforation, multiple intestinal polyps, toxic megacolon or colorectal cancer, etc.
6. Mental disorders or other causes which result in inability of proper expression of feelings.
7. Have participated in other medical clinical studies in the last 3 months.
8. In addition to mesalazine, receiving other treatments for UC.
9. Serious and unstable condition requiring emergency treatment.
10. Inappropriate to participate in this trial judged by clinical physicians.

Termination and withdrawal criteria:

1. The participant is not willing to continue the clinical trial.
2. No medication or any follow-up records;
3. Important deviations occur in the process of the clinical trial, such as poor compliance.
4. Occurrence of allergic reactions or serious adverse events during the trial.
5. Not alleviated or the symptoms are aggravated.

### 2.6 Randomization and Blindness

Institute of Clinical Pharmacology of Xiyuan Hospital, China Academy of Chinese Medical Sciences took the responsibility of generating the allocation sequence. Consecutive numbers were assigned to each clinical center according to a center-stratified random order generated by SAS9.4 (SAS Institute Inc., Cary, NC), and an interactive voice-response or web-response system will be used to assign each patient a number and group. A researcher who will not participate in this study set up an emergency letter for each blinding number, and emergency letters will be sealed in opaque envelopes. In patients with serious adverse events, an emergency unblinding procedure will be initiated. Meanwhile, the whole process of drug coding and documentation is performed blinded. Participants, clinical investigators, and statisticians will all be blinded until completion of the whole trial.

### 2.7 Research personnel

Experienced physicians will be responsible for recruiting and evaluating the participates in this trial. Postgraduates in gastroenterology will be trained by the principal investigator before participating in this trial. They will dispatch drugs, fill in case report forms (CRFs), and record detailed reasons for the patient's withdrawal from the trial.

### 2.8 Recruitment and Encouragement

Six trained investigators and their postgraduates will perform this procedure together. A total 240 patients will be recruited from out-patient and ward of the 6 clinic centers mentioned above, from May 2019 until December 2021. Before the enrollment, all the patients will receive a comprehensive view of informed consent, including objective, therapeutic interventions, scheduling, trial benefits, and possible risks of this trial. Only after signing a written informed consent, patients could be enrolled in it. Meanwhile, to guarantee the completeness of this trial, we will handsel drugs to the patients who have participated it completely.

### 2.9 Collection of biological specimens

The patient's stool, blood and colorectal mucosa samples will be collected before and after intervention, and clearly marked according to the unified format. A designated investigator will centrifuge blood samples in the laboratory. Then, the samples were transferred in frozen pipe and stored at −80°C for preservation in the biological sample bank of School of Life Sciences, Beijing University of Chinese Medicine. Consents and other documents like the collection and usage of participant data and biological specimens could be inquired from the registry web or the corresponding author.

## 3 Interventions

### 3.1 Medications

HEC (HEC, 0.4 g/capsule, supplied by Anhui Joyfar Pharmaceutical, Anhui, China.), EMT (0.5 g/tablet, supplied by LOSAN Pharma GmbH, Neuenburg, Germany),and placebos prepared identical in size, color, shape, taste, smell, and consistency to the 2 above drugs, respectively (supplied by Anhui Joyfar Pharmaceutical, Anhui, China). HEC is a combination of Polygonum cillinerve Ohwi (Zhushaqi), Polygonum Cuspidate (Huzhang), Oldenlandia diffusa (Baihuasheshecao), Sonchus Brachyotus DC (Beibai’jiang), Limonium bicolor (Er'sebuxuecao), Radix Sanguisorbae Preparata Nigra (Diyutan), Bletilla striata (Baiji) and Radix Glycyrrhizae (Gancao). In this trial, an administrator was assigned to manage the drugs, including drug storage, distribution, and recycling independently and kept detailed records.

### 3.2 Intervention plan

Both 2 groups will be treated with drugs for 8 weeks continuously, and the administration method of drugs has been designed as described in Table 1.

**Table 1:**
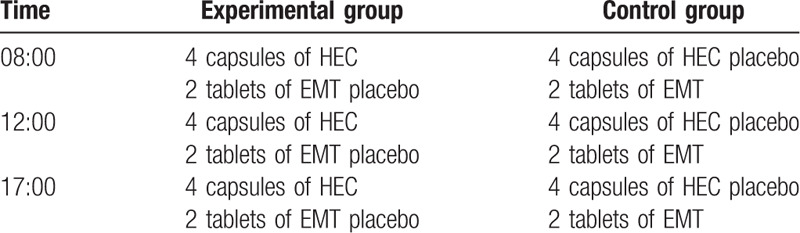
Administration methods in the experimental and control groups.

### 3.3 Follow-up

**Figure 2:**
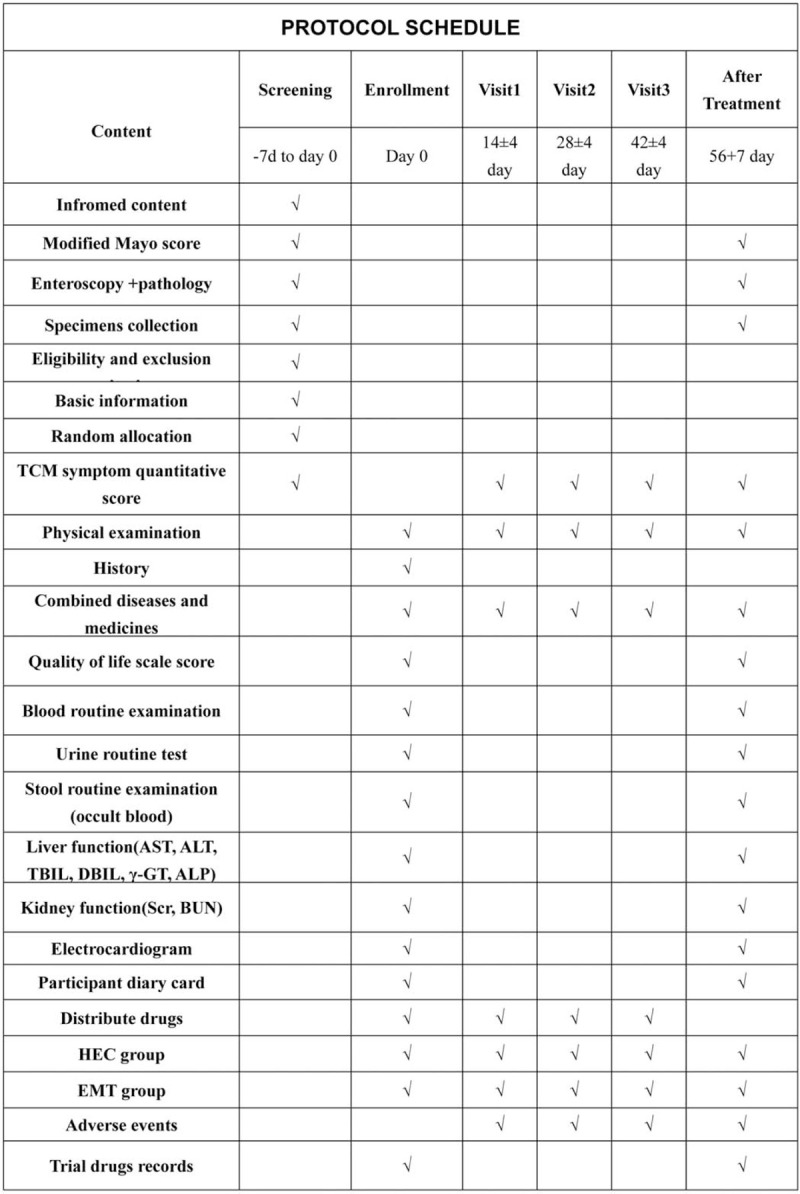
Follow-up protocol schedule chart during the trial. UC = ulcerative colitis, TCM = traditional Chinese medicine.

Each patient was provided with a participant diary card to record diary signs and symptoms of diarrhea, mucilage or blood–pus stools, abdominal pain, and tenesmus. Patients will be interviewed every 2 weeks after enrollment in the trial, while they should return the remaining drugs and packages, and receive the drugs they need to take for the next 2 weeks. Assessment of study endpoints and duration of follow-up was shown in Figure 2.

### 3.4 Outcomes

Primary therapeutic outcome: the rate of clinical efficacy and clinical remission at 8 weeks of treatment (last survey point) according to the modified Mayo score (Table 2). Clinical efficacy was defined as a decrease of at least 30% from the baseline and a decrease ≥3 points, with an accompanying decrease in the subscore for bloody stools ≥1 point or absolute bloody stools subscore of 0 to 1. Clinical efficacy rate = effective number / total number of the group ×100%. Clinical remission was defined as a score ≤2, with no individual subscore with a value greater than 1. Clinical remission rate = remission number / total number of the group ×100%.

**Table 2:**
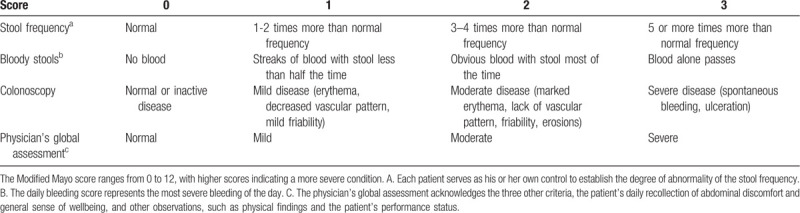
Modified Mayo score system for assessment of ulcerative colitis activity.

Minor outcomes including individual symptom score, TCM syndrome score, endoscopic response rate, mucosal healing rate, and quality of life scale score.

1. Individual symptom score: referred Guidance Principle of Clinical Study on New Drug of Traditional Chinese Medicine.^[17]^ All symptoms were divided into four levels: none, mild, moderate, and severe. The main symptoms were scored as 0, 2, 4, and 6 points, and the minor symptoms are scored as 0, 1, 2, and 3 points. The tongue and pulse were divided into normal and abnormal grades, 0 and 2 points were recorded in the main symptom, and 0 and 1 points were recorded in the minor symptom.
2. TCM syndrome score: The score was graded based on the degree of individual symptom score. All indicators were determined by comparing the values obtained at 2, 4, and 8 weeks with baseline values.
  Clinical recovery: clinical symptoms disappeared or syndrome score decreased by ≥95% from the baseline;
  Marked efficacy: the syndrome score decreased by ≥70% and <95% compared with the baseline;
  Efficacy: the syndrome score was a decrease of at least 30% and less than 70% from the baseline;
  Invalid: the syndrome score was a decrease of less than 30% from the baseline;
  Worsen: the syndrome score exceeded the baseline after treatment.
  TCM syndrome efficacy rate = (Clinical recovery + Marked efficacy + Efficacy) cases /a total number of cases ×100%.
  Endoscopic response rate: was defined as endoscopic score decreased at least 1 point from the baseline in the modified Mayo score. Endoscopic response rate = endoscopic response cases / total number of the group ×100%.
(2) Mucosal healing rate: was defined as absolute subscore for endoscopy of 0 or 1. Mucosal healing rate = Mucosal healing cases / total number of the group ×100%.
(3) Quality of life assessment: Patients were evaluated by Inflammatory Bowel Disease Questionnaire,^[18]^ included the 32 most frequent and important items. Inflammatory Bowel Disease Questionnaire was divided into 4 modules: bowel symptoms (10 questions), systemic symptoms (5 questions), emotional function (12 questions), and social function (5 questions). Each question has graded responses from 1 to 7, and thus the total score is ranging from 32 to 224, with higher scores representing a better quality of life.

### 3.5 Safety assessment

Adverse events (AEs) will be documented and assessed by the local principal investigators during this trial, including clinical signs, symptoms, and laboratory tests. All patients will undergo the following laboratory texts between the start and end of the trial. The safety assessment indices included blood pressure, respiration rate, heart rate, body temperature, and physical examination in addition to blood routine examination, urine routine test, stool routine examination and occult blood, liver function tests (AST, ALT, TBIL, DBIL, γ-GT, ALP), kidney function tests (Scr, BUN) and electrocardiogram. Any AEs will be requested to record in electronic CRFs (eCRFs) regardless of their relationship to the intervention. Severe AEs will be stopped immediately and reported to the local drug administration authorities within 24 hours including a detailed description of the time, severity, possible relationship with the drug, and the treatment measurement will be recorded.

### 3.6 Data management and quality control

The quality assessment of the trial will be conducted in the following aspects: the CRFs, patient diary card, original laboratory examination, informed consent, and drug distribution and storage. The trial information of patient will be recorded in CRFs by a trained and qualified investigator, and the completed CRF will not be allowed to any corrections. The CRFs of each center will be reviewed regularly by clinical inspectors for data quality inspection. The investigators will input and manage the electronic CRFs (eCRFs) online through the Clinical data management system (eCDMS3.0) of Xiyuan Hospital, China Academy of Chinese Medical Sciences. Data input was repeated by 2 investigators to ensure the data accuracy. In addition, all paper documents will be preserved in the Jiangsu Provincial Hospital of Chinese Medicine for 5 years after trial completing and only can be viewed by the research team. Besides, all the datasets will be conducted and monitored by Guangzhou Hipower Pharmaceutical R&D Co, Ltd, which acted as Composition of data monitoring committee of this trial. It will audit the trial conduct and datasets every week.

### 3.7 Data analysis

Data analysis will be performed by professional statisticians and the main researchers using SPSS 22.0. Statistical analysis included the actual number of subjects selected, cases of shedding and rejection, demography and other baseline characteristics, patient compliance, efficacy and safety analysis. We used frequency tables or percentages for categorical variables, and mean ± standard deviation for continuous variables to describe the characteristics of patients in both groups. Categorical data will be analyzed by chi-square test or Fisher exact test to compare the differences between the 2 groups, while quantitative data with normal distribution will be analyzed by *t* test. If the data does not conform to the normal distribution or the uniformity of variance, Wilcoxon rank sum test or Wilcoxon signed test will be used to analyze them to compare the two groups. A two-sided *P-*value <.05 will be set as the significant level.

## 4 Discussion

Compared with UC in Western countries, UC in China has some differences in clinical characteristics. And the prevalence and incidence rates of UC in China were increased rapidly.^[19]^ It is of great significance to find Chinese medicine preparations for the treatment of UC to supplement the selection of clinical drugs. UC is a chronic IBD characterized by a relapsing-remitting course owing to recurrent intestinal inflammation. If the disease can be effectively controlled during mild to moderate period, which will greatly reduce the patient's pain and anxiety. In a clinical randomized controlled trial of HEC in the treatment of active UC, we found the efficacy of TCM syndromes of HEC group was 91.09%, and the positive control group (Mesalazine) was 84.62% after 6 weeks of treatment. HEC was effective in improving the symptoms of patients with mild to moderate UC.^[10]^ The shortcomings of the study were the short study time, lack of enteroscopy, and the Mayo score cannot be used as the primary therapeutic outcome. Therefore, this study extended the treatment time to 8 weeks, completed colonoscopy and intestinal mucosa biopsy, and evaluated the efficacy from the modified Mayo score before and after treatment. The results from this study may provide evidence-based clinical evidence on the effectiveness, safety, and reduce or substitute mesalazine of HEC.

## Acknowledgments

We are grateful to all the researchers and patients who have been involved in this trial.

## Author contributions

**Conceptualization:** Jun-Xiang Li

**Project administration:** Jun Dong, Jun-Xiang Li

**Investigation:** Xiao Ke, Bei-Ping Zhang, Xiao-Jun Yang, Xin-Li Wen, Wei-Yang Zheng, Jun Dong

**Formal analysis:** Xiao Han, Pang-Hua Ding

**Methodology:** Zhi-Bin Wang, Rui Shi, Tang-You Mao

**Writing – original draft:** Fu-Shun Kou, Lei Shi

**Writing – review & editing:** Lei Shi, Fu-Shun Kou, Jun-Xiang Li
